# Male baldness; association with coronary artery disease?

**DOI:** 10.1007/s12471-015-0687-4

**Published:** 2015-04-01

**Authors:** E.E. van der Wall

**Affiliations:** Netherlands Society of Cardiology/Holland Heart House, Moreelsepark 1, 3511 EP Utrecht, The Netherlands

In the literature, there have been conflicting reports on an association between male baldness (androgenetic alopecia) and the incidence of coronary artery disease (CAD).

In 1979, Cooke [1] studied the prevalence of male pattern alopecia, CAD, hypertension, and smoking habits in 478 male Caucasian hospital inpatients over the age of 20 years. No association was shown between CAD and either male pattern alopecia, premature male pattern alopecia, or male pattern alopecia with a positive family history of CAD. In 1990, Herrera et al. [2] identified eight articles containing data on both baldness and CAD. Three of these articles were case-control studies that showed a positive relationship between baldness and CAD, when controlling for CAD risk factors. Three other case-control studies showed no such relationship, but these did not control for CAD risk factors. The results of two cohort studies were inconclusive. Insufficient data were available from these studies to analyze possible relationships between baldness and CAD risk factors themselves. Overall, the data suggested that a small risk of CAD due to baldness might exist, but this risk is much smaller than that of well-known CAD risk factors such as smoking and hypertension [3].

In 2001, Rebora et al. [4] performed a review of 24 articles in the literature from 1954 to 1999 as provided by MEDLINE. Baldness did not coincide with androgenetic alopecia in several of the articles examined, which makes it difficult to settle the issue. Subjects who develop baldness before their 30s may have a higher risk for CAD than other men, and there may be individuals with early-onset androgenetic alopecia who also present with particularly elevated dihydrotestosterone–testosterone ratios. The baldness theory should be included as a secondary hypothesis in large epidemiological studies of CAD.

These predominantly inconclusive studies from the previous century are counterbalanced by several other positive studies performed in the current century. In 2000, Lotufo et al. [5] showed, in a retrospective cohort study among 22,071 US male physicians aged 40–84 years enrolled in the Physicians’ Health Study, that vertex pattern baldness appeared to be a marker for increased risk of coronary events, especially among men with hypertension or high cholesterol levels. In 2001, Matilainen et al. [6] tested if the early onset of alopecia was a risk factor for early CAD in 85 men requiring coronary revascularization, and if the early onset of androgenic alopecia differed in this respect from the late onset of male baldness. The adjusted odds ratio for the coronary revascularization procedure at any age was 1.84 in the subgroup of the men with early androgenetic alopecia compared to those with late androgenic alopecia or normal hair status. These results support the hypothesis that the early onset of androgenic alopecia is a risk factor for an early onset of severe CAD.

Interestingly, in 2005 Mansouri et al. [7] found an association between androgenetic alopecia and CAD in women. The study was carried out in 106 women under the age 55, who underwent coronary arteriography to diagnose CAD. The correlation of androgenetic alopecia and CAD, androgenetic alopecia and a previous history of myocardial infarction, and greying of hair and CAD were statistically significant after adjustment of data for differences in age. These data support the hypothesis that female androgenetic alopecia, like male pattern baldness, is associated with CAD in women under the age 55.

Recently, two studies from the same group showed that early onset androgenetic alopecia in males was independently associated with CAD [8, 9]. In 2013, Sharma et al. [8] performed a comparative study of CAD risk factors in 100 males with androgenetic alopecia grade II between the age of 25 and 40 years with an equal number of age- and sex-matched controls. Patients with androgenetic alopecia appeared to be at an increased risk of developing CAD; therefore, clinical evaluation of cases with androgenetic alopecia grade II and beyond may be of help in preventing CAD in future. Finally, in 2014, the same group investigated the prevalence of androgenetic alopecia in 424 young (< 45 years) Asian Indian Gujarati male patients [9]. A statistically significant association was found between high-grade baldness (Grades IV-VII) and CAD in young men (*p* < 0.05). This study implies that early-onset androgenetic alopecia in males is independently associated with CAD, though the mechanisms need to be investigated.

In the current issue of the Netherlands Heart Journal, Sari et al. [10] investigated the association between male pattern baldness and angiographic severity of CAD and collateral development. Coronary arteriograms, CAD risk factors, lipid parameters, and presence and severity of baldness were prospectively evaluated in 511 male patients. Baldness was classified into five groups. Severity of CAD was evaluated with the Gensini score and collateral development with the Rentrop score, being important measures of presence and severity of CAD. Bald patients had higher Gensini scores when compared with their nonbald counterparts. In univariate analysis, age over 60 years, body mass index more than 30, smoking, and baldness were predictors of high Gensini scores. However, in multivariate analysis only age over 60 years, body mass index more than 30, and smoking were independent predictors of high Gensini scores. No differences were observed in presence and severity of baldness in subjects with and without adequate collateral development. Consequently, the authors did not observe a significant relation between presence, severity, and age of the occurrence of male pattern baldness and severity of CAD.

The present study by Sari et al. [10] provides important data as it contradicts recent studies. The study clearly argues against a significant relationship between androgenetic alopecia and CAD; moreover, Sari et al. are the first to investigate the association between male pattern baldness and angiographic CAD severity and collateral development. In addition, the study provided interesting data such as presence, severity and age of occurrence of male pattern baldness, associated hypertension, and hyperlipidaemia. Of course, as also recognized by the authors, the study has the limitation of having a cross-sectional design. Prospective studies would provide more valuable information to draw conclusions about the occurrence of clinical coronary events in relation to androgenetic alopecia.

Altogether, it goes too far to say that bald is beautiful, but for many men it might be reassuring that male baldness is not significantly associated with CAD.
